# Molecular dynamics simulations and drug discovery

**DOI:** 10.1186/1741-7007-9-71

**Published:** 2011-10-28

**Authors:** Jacob D Durrant, J Andrew McCammon

**Affiliations:** 1 Department of Chemistry and Biochemistry, University of California San Diego, La Jolla, CA 92093, USA; 2 Department of Chemistry and Biochemistry, NSF Center for Theoretical Biological Physics, National Biomedical Computation Resource, University of California San Diego, La Jolla, CA 92093, USA; 3 Department of Pharmacology, University of California San Diego, La Jolla, CA 92093, USA; 4 Howard Hughes Medical Institute, University of California San Diego, La Jolla, CA 92093, USA

**Keywords:** molecular dynamics simulations, computer-aided drug discovery, cryptic binding sites, allosteric binding sites, virtual screening, free-energy prediction

## Abstract

This review discusses the many roles atomistic computer simulations of macromolecular (for example, protein) receptors and their associated small-molecule ligands can play in drug discovery, including the identification of cryptic or allosteric binding sites, the enhancement of traditional virtual-screening methodologies, and the direct prediction of small-molecule binding energies. The limitations of current simulation methodologies, including the high computational costs and approximations of molecular forces required, are also discussed. With constant improvements in both computer power and algorithm design, the future of computer-aided drug design is promising; molecular dynamics simulations are likely to play an increasingly important role.

## Introduction

Richard Feynman, recipient of the 1965 Nobel Prize in Physics, once famously stated: 'If we were to name the most powerful assumption of all, which leads one on and on in an attempt to understand life, it is that all things are made of atoms, and that everything that living things do can be understood in terms of the jigglings and wigglings of atoms.' Much of the biophysics of the last 50 years has been dedicated to better understanding the nature of this atomic jiggling and wiggling. The quantum-mechanical laws governing motions in the microscopic world are surprisingly foreign to those familiar with macroscopic dynamics. Motions are governed not by deterministic laws, but by probability functions; chemical bonds are formed not mechanically, but by shifting clouds of electrons that are simultaneously waves and particles. As Feynman eloquently put it, this is 'nature as she is - absurd' [1].

Understanding these absurd molecular motions is undoubtedly germane to drug discovery. The initial 'lock-and-key' theory of ligand binding [2], in which a frozen, motionless receptor was thought to accommodate a small molecule without undergoing any conformational rearrangements, has been largely abandoned in favor of binding models that account not only for conformational changes, but also for the random jiggling of receptors and ligands [3-7].

**Figure 1:**
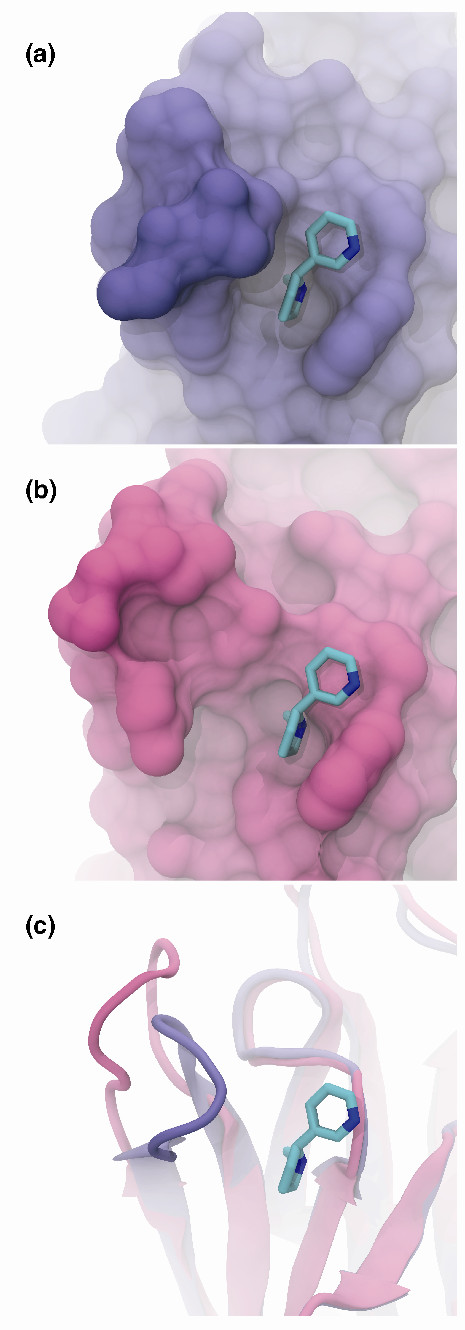
**The different conformations of the acetylcholine binding protein from *Lymnaea stagnalis***. Portions of the protein have been removed to facilitate visualization. **(a) **The protein in a closed conformation with nicotine bound (PDB ID: 1UW6), shown in blue. **(b) **The protein in an open conformation (PDB ID: 1YI5) with the same nicotine conformation superimposed on the structure, shown in pink. **(c) **Ribbon representations of the two conformations.

The mollusk acetylcholine binding protein (AChBP), a structural and functional surrogate of the human nicotinic acetylcholine receptor (AChR) ligand-binding domain [8-11], is one of countless examples that illustrate the importance of accounting for these atomistic motions (Figure 1). In crystallographic structures of small-molecule AChR agonists bound to AChBP, a key loop (loop C) partially closes around the ligand (Figure 1a,c). In contrast, crystal structures of large AChR antagonists like snake α-neurotoxins bound to AChBP reveal that this same loop is displaced by as much as 10 Å, producing an active site that is far more open (Figure 1b,c) [12]. Bourne *et al. *[12] proposed that the unbound AChBP and AChR are highly dynamic proteins that, in the absence of a ligand, sample many conformational states, both open and closed, that are selectively stabilized by the binding of agonists and antagonists. Any one of these binding-pocket conformations may be druggable and therefore pharmacologically relevant. If this general model of ligand binding is correct, the implications for structure-based drug design are profound, as the same principle of binding likely applies to many other systems as well.

## Molecular dynamics simulations

**Figure 2:**
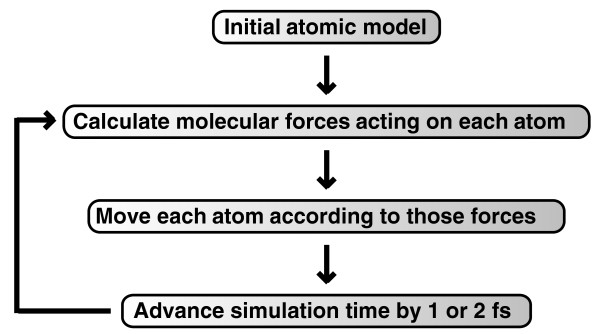
**A schematic showing how a molecular dynamics simulation is performed**. First, a computer model of the receptor-ligand system is prepared. An equation like that shown in Figure 3 is used to estimate the forces acting on each of the system atoms. The positions of the atoms are moved according to Newton's laws of motion. The simulation time is advanced, and the process is repeated many times. This figure was adapted from a version originally created by Kai Nordlund.

**Figure 3:**
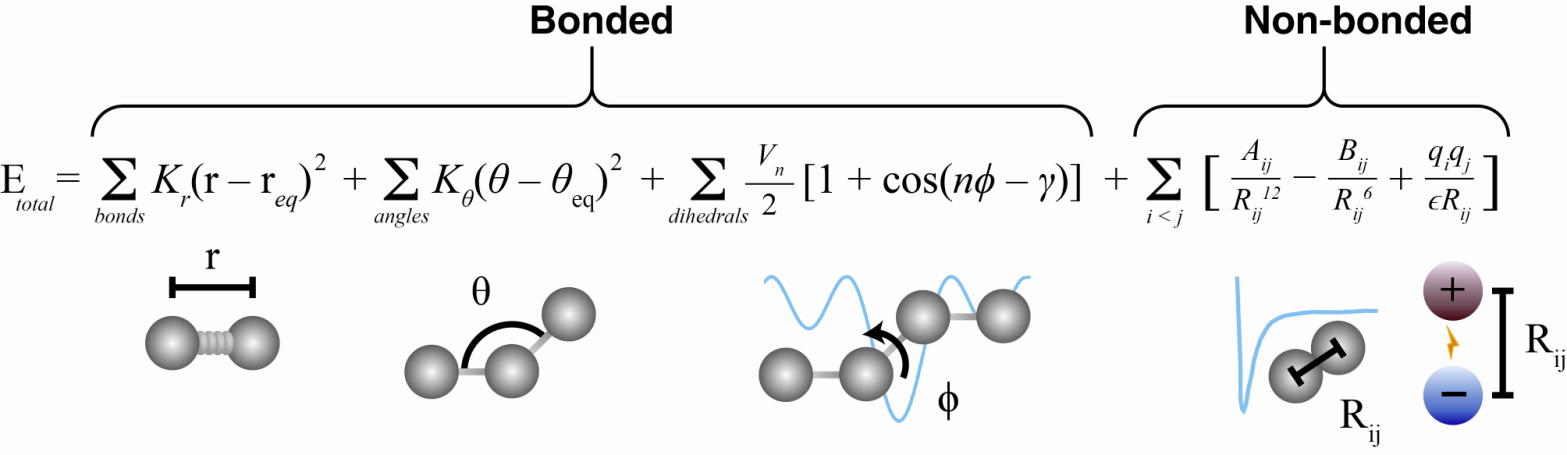
**An example of an equation used to approximate the atomic forces that govern molecular movement**. The atomic forces that govern molecular movement can be divided into those caused by interactions between atoms that are chemically bonded to one another and those caused by interactions between atoms that are not bonded. Chemical bonds and atomic angles are modeled using simple springs, and dihedral angles (that is, rotations about a bond) are modeled using a sinusoidal function that approximates the energy differences between eclipsed and staggered conformations. Non-bonded forces arise due to van der Waals interactions, modeled using the Lennard-Jones potential, and charged (electrostatic) interactions, modeled using Coulomb's law.

While crystallographic studies like these convincingly demonstrate the important role protein flexibility plays in ligand binding, the expense and extensive labor required to generate them have led many to seek computational techniques that can predict protein motions. Unfortunately, the calculations required to describe the absurd quantum-mechanical motions and chemical reactions of large molecular systems are often too complex and computationally intensive for even the best supercomputers. Molecular dynamics (MD) simulations, first developed in the late 1970s [13], seek to overcome this limitation by using simple approximations based on Newtonian physics to simulate atomic motions, thus reducing the computational complexity. The general process of approximation used is outlined in Figure 2. First, a computer model of the molecular system is prepared from nuclear magnetic resonance (NMR), crystallographic, or homology-modeling data. The forces acting on each of the system atoms are then estimated from an equation like that shown in Figure 3[14]. In brief, forces arising from interactions between bonded and non-bonded atoms contribute. Chemical bonds and atomic angles are modeled using simple virtual springs, and dihedral angles (that is, rotations about a bond) are modeled using a sinusoidal function that approximates the energy differences between eclipsed and staggered conformations. Non-bonded forces arise due to van der Waals interactions, modeled using the Lennard-Jones 6-12 potential [15], and charged (electrostatic) interactions, modeled using Coulomb's law. For a more in-depth review describing how the equations describing these interactions are parameterized, see [14].

In order to reproduce the actual behavior of real molecules in motion, the energy terms described above are parameterized to fit quantum-mechanical calculations and experimental (for example, spectroscopic) data. This parameterization includes identifying the ideal stiffness and lengths of the springs that describe chemical bonding and atomic angles, determining the best partial atomic charges used for calculating electrostatic-interaction energies, identifying the proper van der Waals atomic radii, and so on. Collectively, these parameters are called a 'force field' because they describe the contributions of the various atomic forces that govern molecular dynamics. Several force fields are commonly used in molecular dynamics simulations, including AMBER [14,16], CHARMM [17], and GROMOS [18]. These differ principally in the way they are parameterized but generally give similar results.

Once the forces acting on each of the system atoms have been calculated, the positions of these atoms are moved according to Newton's laws of motion. The simulation time is then advanced, often by only 1 or 2 quadrillionths of a second, and the process is repeated, typically millions of times. Because so many calculations are required, molecular dynamics simulations are typically performed on computer clusters or supercomputers using dozens if not hundreds of processors in parallel. Many of the most popular simulation software packages, which often bear the same names as their default force fields (for example AMBER [19], CHARMM [17], and NAMD [20,21]), are compatible with the Message Passing Interface (MPI), a system of computer-to-computer messaging that greatly facilitates the execution of complex tasks by one software application on multiple processors operating simultaneously.

While the utility of molecular dynamics simulations should not be overstated, a number of studies comparing these simulations with experimental data have been used to validate the computational technique [22]. NMR data are particularly useful, as the many receptor and ligand conformations sampled by molecular dynamics simulations can be used to predict NMR measurements like spin relaxation, permitting direct comparison between experimental and theoretical techniques. Indeed, a number of studies have shown good agreement between computational and experimental measurements of macromolecular dynamics [23-26].

## Molecular dynamics simulations: current limitations

These successes aside, the utility of molecular dynamics simulations is still limited by two principal challenges [27]: the force fields used require further refinement, and high computational demands prohibit routine simulations greater than a microsecond in length, leading in many cases to an inadequate sampling of conformational states. As an example of these high computational demands, consider that a one-microsecond simulation of a relatively small system (approximately 25,000 atoms) running on 24 processors takes several months to complete.

Aside from challenges related to the high computational demands of these simulations, the force fields used are also approximations of the quantum-mechanical reality that reigns in the atomic regime. While simulations can accurately predict many important molecular motions, these simulations are poorly suited to systems where quantum effects are important, for example, when transition metal atoms are involved in binding.

To overcome this challenge, some researchers have introduced quantum mechanical calculations into classic molecular-dynamics force fields; the motions and reactions of enzymatic active sites or other limited areas of interest are simulated according to the laws of quantum mechanics, and the motions of the larger system are approximated using molecular dynamics. While far from the computationally intractable 'ideal' of using quantum mechanics to describe the entire system, this hybrid technique has nevertheless been used successfully to study a number of systems. For example, in one recent simulation of *Desulfovibrio desulfuricans *and *Clostridium pasteurianum *[Fe-Fe] hydrogenases, a 'QM [quantum mechanical] region' was defined encompassing a metal-containing region of the protein thought to be catalytically important, and the remainder of the protein was simulated using classical molecular dynamics [28]. The simulations revealed an important proton transfer in the QM region, a bond-breaking and bond-formation event that could not have been modeled with a traditional force field. The hypothesized catalytic mechanism was subsequently supported by experimental evidence.

Aside from bond breaking and formation, electronic polarization, caused by the flow of electrons from one atomic nucleus to another among groups of atoms that are chemically bonded, is another quantum-mechanical effect that, with few exceptions, is generally ignored. In classical molecular dynamics simulations, each atom is assigned a fixed partial charge before the simulation begins. In reality, however, the electron clouds surrounding atoms are constantly shifting according to their environments, so that the partial charges of atoms would be better represented as dynamic and responsive. Despite wide agreement on the importance of accounting for electronic polarization, after 30 years of development a generally accepted polarizable force field has not been forthcoming, and molecular dynamics simulations using those force fields that are available are rare [29]. Nevertheless, a number of polarizable force fields are currently under development [30], and future implementations may lead to improved accuracy.

In addition to neglecting quantum-mechanical effects, molecular dynamics studies are also limited by the short time scales typically simulated. To reproduce thermodynamic properties and/or to fully elucidate all binding-pocket configurations relevant to drug design, all the possible conformational states of the protein must be explored by the simulation. Unfortunately, many biochemical processes, including receptor conformational shifts relevant to drug binding, occur on time scales that are much longer than those amenable to simulation. With some important exceptions [31], simulations are currently limited to at most millionths of a second; indeed, most simulations are measured in billionths of a second.

A number of solutions to this challenge have already seen limited use. For example, in accelerated molecular dynamics (aMD) [32,33], large energy barriers are artificially reduced. Though this process inevitably introduces some artifacts, it does allow proteins to shift between conformations that would not be accessible given the time scales of conventional molecular dynamics. These novel conformations can then be further studied using classical molecular dynamics or other techniques.

Novel hardware has also been used to overcome the time-scale limitations of conventional molecular dynamics simulations. Many of the same calculations required for these simulations are commonly performed by video-game and computer-graphics applications. Consequently, the same graphics-processing-units (GPUs) designed to speed up video games can be used to speed up molecular dynamics simulations as well, usually by an order of magnitude [34-36].

Not satisfied with merely adapting molecular-dynamics code to run on specialized graphics processors, some engineers have designed new processors specifically for these simulations. The research group of DE Shaw is one notable advocate of this approach. They have built a supercomputer codenamed Anton capable of performing microseconds of simulation per day. With Anton, simulations longer than one millisecond [31] have successfully captured protein folding and unfolding as well as drug-binding events [37]. Shortcomings certainly still exist, but these and other future techniques will likely make great progress towards overcoming current limitations on conformational sampling.

## Molecular dynamics simulations and drug discovery

Weaknesses in current force fields and conformational sampling aside, molecular dynamics simulations and the insights they offer into protein motion often play important roles in drug discovery. Just as a single photograph of a runner tells little about her stride, a single protein conformation tells little about protein dynamics. The static models produced by NMR, X-ray crystallography, and homology modeling provide valuable insights into macromolecular structure, but molecular recognition and drug binding are very dynamic processes. When a small molecule like a drug (for example, a ligand) approaches its target (for example, a receptor) in solution, it encounters not a single, frozen structure, but rather a macromolecule in constant motion.

In some rare cases, protein motions are limited, and the ligand may fit into a fairly static binding pocket like a key fits into a lock [2]. More typically, the ligand may bind and stabilize only a subset of the many conformations sampled by its dynamic receptor, causing the population of all possible conformations to shift towards those that can best accommodate binding [4-7]. Upon binding, the ligand may further induce conformational changes that are not typically sampled when the ligand is absent [38]. Regardless, receptor motions clearly play an essential role in the binding of most small-molecule drugs. Several techniques have been developed to exploit the information about these motions that molecular dynamics simulations can provide.

## Identifying cryptic and allosteric binding sites

NMR and X-ray crystallographic structures often reveal well defined binding pockets that accommodate endogenous ligands; however, sometimes the models produced by these experimental techniques obscure other potentially druggable sites. As these sites are not immediately obvious from available structures, they are sometimes called cryptic binding sites.

Molecular dynamics simulations are excellent tools for identifying such sites [39-41]. For example, in 2004 Schames *et al. *[39] performed a molecular dynamics simulation of HIV integrase, a drug target that had not seemed amenable to structure-based drug design. The simulations revealed a previously unidentified trench that was not evident from any of the available crystal structures. X-ray crystallography later demonstrated that known inhibitors do in fact bind in this cryptic trench, as predicted. These results led to new experimental studies at Merck & Co. [42]; further development ultimately resulted in production of the highly effective antiretroviral drug raltegravir, the first US Food and Drug Administration-approved HIV integrase inhibitor.

Aside from cryptic binding sites, molecular dynamics simulations can also be used to identify druggable allosteric sites. In one recent study, Ivetac and McCammon [43] performed simulations of the human β_1 _(β_1_AR) and β_2 _(β_2_AR) adrenergic receptors. Multiple protein conformations were extracted from these simulations, and the protein surface was computationally 'flooded' with small organic probes using FTMAP [44] to identify potential binding sites. Regions of the protein surface where the organic probes consistently congregated across multiple structures were then identified as potential allosteric sites. In all, five potential sites were identified, some of which were not evident in any of the existing crystal structures.

## Improving the computational identification of true small-molecule binders: the relaxed complex scheme

One common technique used to identify the precursors of potential drugs *in silico *is virtual screening. A docking program is used to predict the binding pose and energy of a small-molecule model within a selected receptor binding pocket. Traditionally, many ligand models, typically taken from a database of compounds that can be easily synthesized or commercially purchased, are docked into a single static receptor structure, often obtained from NMR or X-ray crystallography. The best predicted ligands are subsequently tested experimentally to confirm binding.

Unfortunately, traditional docking relying on a single receptor structure is problematic. Some legitimate ligands may indeed bind to the single structure selected, but in reality most receptor binding pockets have many valid conformational states, any one of which may be druggable. In a traditional virtual screen, true ligands are often discarded because they in fact bind to receptor conformations that differ markedly from that of the single static structure chosen.

To better account for receptor flexibility, a new virtual-screening protocol has been developed called the relaxed complex scheme (RCS) [45,46]. Rather than docking many compound models into a single NMR or crystal structure, each potential ligand is docked into multiple protein conformations, typically extracted from a molecular dynamics simulation. Thus, each ligand is associated not with a single docking score but rather with a whole spectrum of scores. Ligands can be ranked by a number of spectrum characteristics, such as the average score over all receptors. Thus, the RCS effectively accounts for the many receptor conformations sampled by the simulations; it has been used successfully to identify a number of protein inhibitors, including inhibitors of FKBP [47], HIV integrase [39], *Trypanosoma brucei *RNA editing ligase 1 [48,49], *T. brucei *GalE [50], *T. brucei *FPPS [51], and *Mycobacterium tuberculosis *dTDP-6-deoxy-L-lyxo-4-hexulose [52]. In two of these projects, the identified inhibitors were effective not only against the target proteins, but against the whole-cell parasite [49,50].

While these successes are promising, the relaxed complex scheme certainly has its weaknesses. Aside from being based on molecular dynamics simulations that are themselves subject to crude force-field approximations and inadequate conformational sampling, the scheme relies on computer-docking scoring functions that of necessity are optimized for speed at the expense of accuracy. In order to facilitate high-throughput virtual screening, these scoring functions often treat subtle influences on binding energy like conformational entropy and solvation energy only superficially [27,53], thus sacrificing accuracy for the sake of greater speed.

## Advanced free-energy calculations using molecular dynamics simulations

Though docking programs are optimized for speed rather than accuracy, more accurate, albeit computationally intensive, techniques for predicting binding affinity do exist. These techniques, which include thermodynamic integration [54], single-step perturbation [55], and free energy perturbation [56], are based in large part on molecular dynamics simulations.

Free energy is a state function, meaning that the free-energy difference associated with a given event like a drug binding to its receptor is determined only by the energy prior to that event and the energy following it; while the path taken from the initial to the final state may influence receptor-ligand kinetics, it has no bearing on the free energy. Perhaps the ligand diffuses slowly towards the active site and slips easily into the binding pocket. Perhaps the protein unfolds entirely and then refolds around the ligand. Perhaps the ligand in solution is beamed to a starship in orbit, only to rematerialize in the active site a few seconds later. The mechanics do not matter; the free energy depends only on the initial energy in solution and the final energy following the binding event.

With some notable exceptions [37], simulating a receptor-ligand system long enough to capture an entire binding event is not currently feasible. However, it is still possible to calculate a drug's binding affinity using a technique called 'alchemical transformation', first described in 1984 [57]. This transformation is not too different from the starship example given above. During the course of a molecular dynamics simulation, the electrostatic and van der Waals forces produced by ligand atoms are turned down gradually enough to avoid undesirable artifacts. Eventually, the ligand is no longer able to interact with the protein or solvent. For all practical purposes, the ligand has disappeared. It does not matter that this transformation is not at all physical; free energy is a state function, so the path from the initial to the final state, whether real or imaginary, is irrelevant.

**Figure 4:**
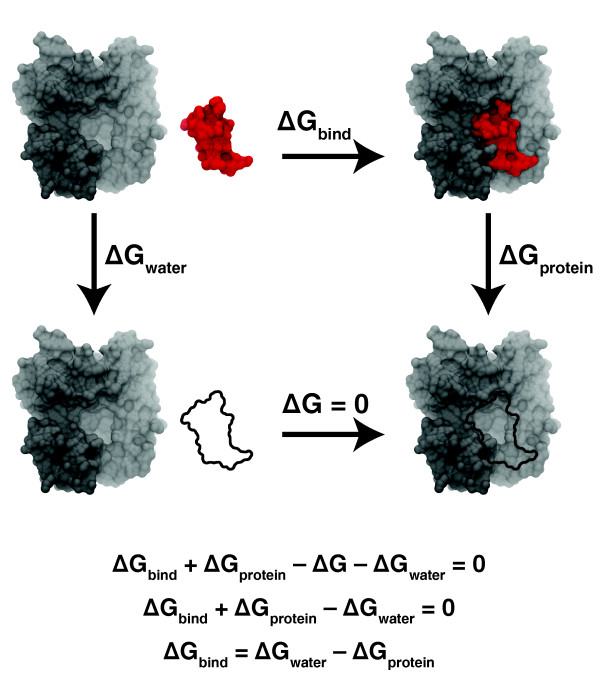
**The thermodynamic cycle used for selecting alchemical transformations**. Typically, one wishes to calculate the free energy of binding, ΔG_bind_, shown across the top. However, it is generally impractical to run a molecular dynamics simulation long enough to capture an entire binding event. Instead, a series of alchemical transformations are performed using molecular dynamics simulations. ΔG_protein _is the change in free energy that occurs when a bound ligand is 'annihilated'. ΔG is the change in free energy that occurs when an unbound 'ghost' ligand binds to the receptor; however, since a ghost ligand is not able to interact with any solvent or receptor atoms, this energy is always zero. Finally, ΔG_water _is the change in free energy that occurs when an unbound ligand in solution is 'annihilated'. A system that proceeds from one state around this free-energy cycle only to return to the same initial state should have no change in total free energy; consequently, ΔG_bind _= ΔG_water _- ΔG_protein_.

It is not clear, however, in what context the ligand should be figuratively annihilated in this way. Should a molecular dynamics simulation be run in which the bound ligand vanishes? What about the ligand in solution? To address these questions, alchemical transformations are selected based on the thermodynamic cycle shown in Figure 4. As free energy is a state function that depends only on the energy of the initial and final states, a system that proceeds from one state around this free-energy cycle only to return to the same initial state should have no change in total free energy (that is, ΔG_bind _+ ΔG_protein _- ΔG - ΔG_water _= 0). We note that ΔG in this equation is itself zero, since the ligand is entirely disappeared in both of the states shown in the bottom half of Figure 4, meaning the ligand cannot interact with the water solvent or the protein in either state. Thus, ΔG_bind _+ ΔG_protein _- ΔG_water _= 0, and, consequently, ΔG_bind _= ΔG_water _- ΔG_protein_. These equations demonstrate that it is possible to estimate a drug's free energy of binding, an indirect measurement of drug potency, by running two simulations, one in which the receptor-bound ligand disappears, and one in which the solvated ligand disappears.

A similar task, calculating relative ligand binding energies, is useful during drug optimization when one wishes to determine if a given chemical change will improve the potency of a candidate ligand. In this case, rather than annihilating the entire ligand, one section of the ligand is gradually transformed. For example, a key carbon atom might be gradually converted into an oxygen atom to see if the binding affinity is improved or diminished. These kinds of alchemical molecular dynamics simulations may provide medicinal chemists with useful insights that can guide further drug development.

A series of early fortuitous results that agreed remarkably well with experiments led many to an enthusiasm for molecular-dynamics-based free-energy calculations in the 1980s and early 1990s that was not matched in subsequent decades [27,58] as computational predictions fell short of experimental measurements. However, steady algorithmic and engineering advances in recent years have led to renewed attention. The successful applications of alchemical techniques in recent years are legion; accurate predictions have been obtained for ligand binding to the src SH2 domain [59], to a mutant T4 lysozyme [60], to FKBP12 [61], to HIV reverse transcriptase [62], to trypsin [63], to a bacterial ribosome [64], and to estrogen receptor-α [65], among many others.

These successes aside, it is important not to oversell alchemical techniques. All molecular-dynamics-based drug-discovery techniques would benefit from improved force fields, but the alchemical techniques are, in addition, uniquely sensitive to inadequate conformational sampling [66]. When molecular dynamics simulations of insufficient length are used to identify cryptic sites, allosteric sites, or pharmacologically relevant binding-pocket conformations for virtual-screening projects, the risk is that some suitable receptor conformations will be missed. The conformations that are identified, however, are still useful; the results of the simulation are therefore incomplete, but not necessarily wrong.

However, the alchemical techniques used to calculate free energies of binding are far more dependent on thorough conformational sampling than are RCS screens. If molecular dynamics simulations fail to sample system conformations that are in fact sampled *ex silico*, these conformations will not contribute to the total calculated energy, leading to an incorrect prediction of the binding affinity. Molecular dynamics simulations are computationally demanding and often of necessity unacceptably short; insufficient conformational sampling is therefore a common problem that future algorithmic and hardware-engineering efforts must address. It is largely because accurate predictions often require lengthy simulations that these alchemical techniques have not yet been widely adopted by the pharmaceutical industry, despite great interest [27].

## Conclusions

In this review, we have discussed the many roles that molecular dynamics simulations can play in drug discovery, including the identification of cryptic or allosteric binding sites, the enhancement of traditional virtual-screening methodologies, and the direct prediction of ligand binding energies. As ligand binding and the important macromolecular motions associated with it are microscopic events that take place in mere millionths of a second, a complete understanding of the atomistic energetics and mechanics of binding is unattainable using current experimental techniques. Molecular dynamics simulations are useful for filling in the details where experimental methods cannot.

With constant improvements in both computer power and algorithm design, the future of computer-aided drug design is promising; molecular dynamics simulations are likely to play an increasingly important role in the development of novel pharmacological therapeutics.
